# Modular MA-XRF Scanner Development in the Multi-Analytical Characterisation of a 17th Century *Azulejo* from Portugal †

**DOI:** 10.3390/s21051913

**Published:** 2021-03-09

**Authors:** Sergio Augusto Barcellos Lins, Marta Manso, Pedro Augusto Barcellos Lins, Antonio Brunetti, Armida Sodo, Giovanni Ettore Gigante, Andrea Fabbri, Paolo Branchini, Luca Tortora, Stefano Ridolfi

**Affiliations:** 1 Dipartimento di Scienze di Base e Applicate per L’ingegneria, Università degli Studi di Roma “La Sapienza”, Via Antonio Scarpa 14/16, 00161 Rome, Italy; giovanni.gigante@uniroma1.it; 2 Surface Analysis Laboratory Roma Tre—National Institute of Nuclear Physics—Roma Tre University, Via della Vasca Navale 84, 00146 Rome, Italy; paolo.branchini@roma3.infn.it (P.B.); luca.tortora@uniroma3.it (L.T.); 3 LIBPhys & VICARTE, Department of Physics & Department of Conservation and Restoration, NOVA School of Science and Technology, 2829-516 Caparica, Portugal; marta.manso@fct.unl.pt; 4 Instituto de Química, Universidade Federal Fluminense, Rua Mario Santos Braga, 30-Centro, Niterói, RJ 24020-140, Brazil; pedrolins@id.uff.br; 5 Istituto di Matematica e Fisica, Università degli Studi di Sassari, Via Vienna 2, 07100 Sassari, Italy; 6 Department of Sciences, Via della Vasca Navale 84, 00146 Rome, Italy; armida.sodo@uniroma3.it; 7 INFN Sezione di Roma Tre, Via della Vasca Navale 84, 00146 Rome, Italy; andrea.fabbri@roma3.infn.it; 8 Ars Mensurae, Via Vincenzo Comparini 101, 00188 Rome, Italy; stefano@arsmensurae.it

**Keywords:** MA-XRF, Monte Carlo simulations, *azulejos*, Raman spectroscopy

## Abstract

A modular X-ray scanning system was developed, to fill in the gap between portable instruments (with a limited analytical area) and mobile instruments (with large analytical areas, and sometimes bulky and difficult to transport). The scanner has been compared to a commercial tabletop instrument, by analysing a Portuguese tile (*azulejo*) from the 17th century. Complementary techniques were used to achieve a throughout characterisation of the sample in a complete non-destructive approach. The complexity of the acquired X-ray fluorescence (XRF) spectra, due to inherent sample stratigraphy, has been resolved using Monte Carlo simulations, and Raman spectroscopy, as the most suitable technique to complement the analysis of *azulejos* colours, yielding satisfactory results. The colouring agents were identified as cobalt blue and a Zn-modified Naples-yellow. The stratigraphy of the area under study was partially modelled with Monte Carlo simulations. The scanners performance has been compared by evaluating the images outputs and the global spectrum.

## 1. Introduction

Decorative ceramic glazed tiles (*azulejos*) are an intrinsic part of Portuguese culture, being widely used in the country to decorate the most varying surfaces since the 15th century until today. Their local use is believed to have started around the 13th century through Moorish influence, while the *majolica* technique arrived in Portugal only later, around the 16th century, with the establishment of local workshops [1,2].

The *azulejos* are mainly composed of a ceramic biscuit and glaze, the latter comprising a wide gamut of colours, which can act as a fingerprint of the *azulejo*, allowing the identification of its origin, period, and manufacture technique [1,3,4,5]. Their stratigraphy and composition can be analysed by sampling and cross-sectioning the artefact, an approach not always feasible or desired. An alternative way to avoid a destructive approach is to perform Monte Carlo simulations of X-ray fluorescence spectra. By iteratively simulating the experimental data, it is possible to model the stratigraphy and composition of the irradiated sample up to a satisfactory degree of accuracy [6,7,8].

When it comes to a complete non-destructive analysis, the glaze and pigments chemical composition can be investigated by X-ray fluorescence (XRF) and Raman spectroscopy [1,4,5]. The XRF punctual analysis, in particular, yields local information, which does not always represent the sample as a whole. In this case, it is convenient to map the artefacts’ surface, gaining information on the spatial distribution of chemical species.

The use of X-ray fluorescence scanners in heritage sciences is becoming a common practice, thanks to the spatial information on chemical elements being more crucial for the analysis of painted surfaces, and the growing popularization and accessibility of these instruments [9,10,11,12,13]. However, it is often the case where these artworks cannot be transported into laboratories or synchrotron facilities due to their valuable nature [14]. This has led to a spike in the development of transportable and, more recently, portable systems [15,16,17]. Yet, the design choice of developing a portable or mobile X-ray fluorescence scanner comes with crucial limitations. A portable unit offers a limited scanning area, while a mobile one provides a much larger scanning area, at the cost of sacrificing its portability.

When developing an *in-house* and accessible scanning system, other parameters, such as pricing, must be taken into consideration. Most of the systems’ cost is related to the X-ray electronics. This cost can be reduced if low-power electronics and simple focusing optics are used, with the drawback often resulting in longer dwell-times and lower resolutions [18,19]. Furthermore, as system portability and maximum scanning area are competing parameters, difficult choices, apart from budget, must be made when considering developing one single scanning system, sacrificing either one or another parameter.

In this scope, combining cost-effectiveness and versatility, a modular scanning unit was developed in a joint effort between Ars Mensurae, *Istituto Nazionale di Fisica Nucleare—Roma TRE* and Sapienza University. The system was put to test against a commercial tabletop μ-XRF scanner. The data collected with both instruments was used, combined with other analytical techniques, to fully characterize a Portuguese *azulejo* belonging to the 17th century.

## 2. Materials and Methods

**Figure 1:**
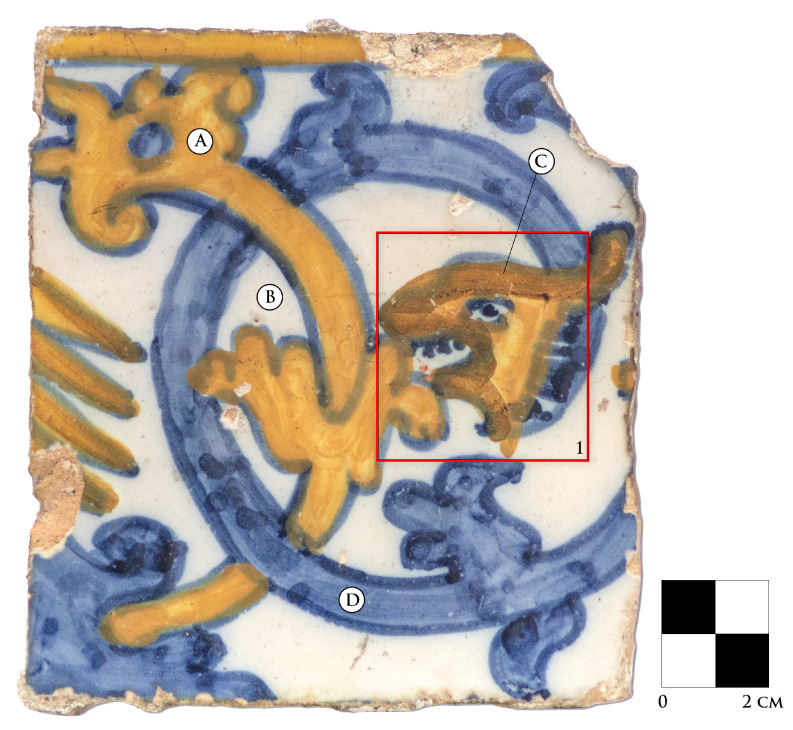
Sample and Macro-X-Ray Fluorescence (MA-XRF) scanned area (1). The colours identified are: yellow (**A**), white (**B**), orange (**C**) and blue (**D**).

A Portuguese tile (*azulejo*) from approximately the 17th century was investigated with the proposed Macro-X-Ray Fluorescence (MA-XRF) scanning system and complementary techniques. The *azulejo* is shown in Figure 1, where four colours can be identified—yellow (A), white (B), orange (C), and blue (D). Area 1 represents the region investigated through MA-XRF scanning.

Monte Carlo simulations of XRF spectra acquired in different spots of the tile were performed to better elucidate the stratigraphy and composition of the yellow, white and blue coloured areas. Furthermore, μ-Raman analysis of several other spots were performed, serving as a complementary technique to better understand the nature of the colouring agents.

### 2.1. MA-XRF Scanning

Elemental distribution maps from a ≈25 cm${}^{2}$ portion of the *azulejo* were acquired with the Modular MA-XRF Scanner. The results were compared with those obtained with a commercial, tabletop scanner from BRUKER^®^ GmbH, Germany. The analysis conditions and system setups, as well as a description of the Modular Scanner, are presented in the following subsections.

#### 2.1.1. System Development

**Figure 2:**
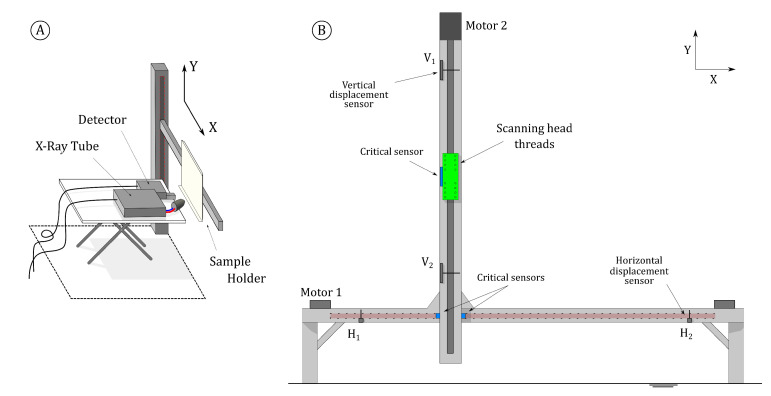
Translation stages: portable version (**A**) and mobile version (**B**).

Figure 2 shows a schematic drawing of the translation stages designed for the scanning system described herein. To overcome one of the major disadvantages of portable systems, two translation stages were developed, one being small and portable, capable of scanning areas up to 20 × 20 cm${}^{2}$, and a larger, mobile one.

Multiple X-rays detectors can be fitted in the scanning head to reach shorter dwell-times if needed. The X-ray tube can be exchanged, as the head threading can fit any of Moxtek’s^®^ analog-handheld tubes. The beam is focused with an exchangeable aluminium collimator. The current prototype iteration uses up to 3 AMPTEK^®^ X-123 Silicon-Drift Detectors (SDD), which are low power and lightweight. The detectors are configured identically, so acquisition can be synchronized and the sum spectrum from all detectors viewed in live time.

In addition to the X-rays electronics, the x-y translation stage can be exchanged as well. This allows one to achieve quick assembly times (ca. 25 min) as well as provides an extra degree of versatility. X-rays detectors and translation stage(s) are controlled by the same acquisition software, developed in the LabVIEW^®^ platform. The tube is controlled externally, not integrated in the controlling software, enforcing the system’s modularity.

In sum, the system can be configured to suit each necessity. The scanning head (with only one detector), cables, small stage and all related peripherals can comfortably fit inside a standard airplane cabin trolley. The portable version is described in more detail elsewhere [20].

#### 2.1.2. Data Acquisition

The areas scanned by both the Modular Scanner and the M4 Tornado are highlighted in Figure 1. Although M4 Tornado can provide considerably shorter dwell-times, thanks to its higher power X-ray tube, both analyses were performed under the most similar conditions possible, to ascertain whether the results are comparable. Scanned areas were 5.3 × 5.0 cm${}^{2}$ in size each and were acquired with a tube voltage of 35 kV and with an applied current of 29 μA. The areas scanned with each instrument were slightly offset from one another and were cropped to improve the comparison and overlay between them.

Dwell-times were set to 500 ms for the M4 Tornado and 470 ms for the Modular Scanner, with an overall stage speed of approximately 2 mm/s. The M4 Tornado scanner is equipped with a Rh target X-ray tube, while the Modular Scanner had an Ag target Moxtek^®^ tube attached. The Modular Scanner was assembled with the mobile stage and two X-123 SDD AMPTEK^®^ detectors.

Overall scanning time was about 20 min for each instrument, with both acquisitions being performed in a continuous scan fashion. Elemental distribution maps were generated by different software. M4 Tornado images were obtained with Bruker ESPRIT proprietary software. Data obtained with the Modular Scanner were processed with an *in-house* developed software (XISMuS) version 1.3.2 [21].

### 2.2. Monte Carlo Simulations

The XRF spectra simulations were carried with a modified version of the XRMC package [22], version 6.4.1., a Monte Carlo algorithm based on the xraylib [23] database. This XRMC version is capable of simulating rough surfaces as well as up to any order of photoelectric interaction. However, for the present purpose, a threshold was set at the 3rd order, since errors in the atomic parameters accumulate as the interaction order increases, and results may lose significance.

Experimental data were collected with a portable XRF spectrometer, from 3 spots within the sample surface, representing the white, yellow, and blue colours. For the blue colour, two spectra were collected, one for the light shade of blue and one for the darker shade, in the right-most part of area 1 (Figure 1). The spectrometer used was composed of a Mini-X tube, from AMPTEK^®^ with a silver anode, collimated to 1 mm and an X-123 SDD X-ray detector, also from AMPTEK^®^. The system geometry was set as the following: detector parallel to the sample’s normal, 3 cm distant and uncollimated; tube at 45 degree from the sample’s normal and 2 cm distant. The tube operated with a voltage of 40 kV and an applied current of 15 μA.

Each simulation result is obtained iteratively, with user intervention at each iteration step. The simulation starts with an initial guess, essentially based on the peaks visible in the measured spectrum and, when possible, on the typical composition of the sample as reported in literature as well. The X-Ray excitation spectrum and geometrical factors of the portable XRF instrument have been carefully modeled using several measurements of reference samples.

The *azulejo* has been modeled as a set of layers, each of them identified by six flat planes (called quadrics in terms of XRMC parameters). The simulated and measured spectra are compared at each iteration, and, if differences are detected, the simulation parameters are adjusted accordingly. These are all significative parameters required to perform a simulation. This protocol has been previously and successfully applied to other archaeological materials, and is described in more detail elsewhere [7,8,24].

### 2.3. μ-Raman Analysis

Raman spectra from 4 different spots, covering all the detected colours, were collected with the InVia™ μ-Raman spectrometer from RENISHAW^®^, UK, equipped with a Leica DM2700 M confocal microscope. The spots were analysed with two different solid-state diode laser sources, one at 532 nm and the second at 785 nm, selected according to need. The former source operated at 120 mW power while the latter operated at 250 mW. Neutral filters were used to decrease the power at the sample, taking into account its nature and composition thus avoiding degradation processes. A set of long working distance objectives (namely 20× and 50×) was used, focusing the excitation beam down to few microns.

To achieve enough statistics, 3 to 5 scans, with an integration time of 5 s were acquired. The data were collected with the instrument’s proprietary software and then exported as ASCII files. The output spectra were processed with a graphic analysis software.

## 3. Results and Discussion

### 3.1. Scanner Performance

**Figure 3:**
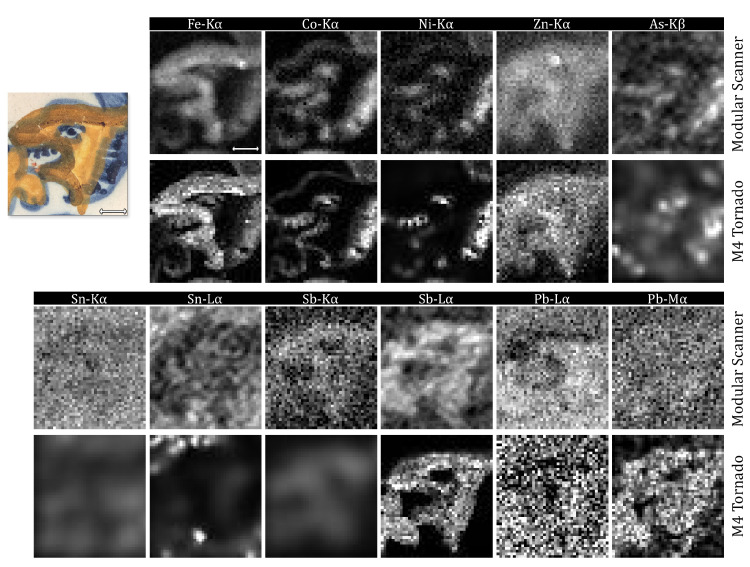
Elemental distribution maps obtained with the Modular Scanner and the M4 Tornado. Scale is 10 millimetres.

The *in-house* developed instrument had its performance compared to a state-of-the-art one, and the results obtained can be seen in Figure 3. It is important to highlight that the optics chosen can play a significant role when it comes to final resolution, as the irradiation spot can vary widely (from few millimetres to few micrometres). In the case of our instrument, an aluminium collimator of 2 mm diameter was used, while the M4 Tornado had a polycapillary lens focusing the beam (at 20 μm for Mo-$K_{\alpha }$ energy and 38 μm for Mo-$L_{\alpha }$ energy). This ensued slightly more blurred images with the Modular Scanner in respect to the M4 Tornado. Significant differences can be observed in all images of As, Sn, Sb, and Pb elemental distribution maps. At first, it is plausible to assume this may be due to the M4 Tornado detector’s resolution and number of channels, the latter 4 times greater than that set at the X-123 SDD detectors in the Modular Scanner, conceiving some significant superior resolving power. Moreover, the narrower beam provided by the M4 Tornado instrument, even though provides an inferior count rate (Supplementary Material Figure S2), allows the collection of more localized information.

The elemental distribution maps were obtained through different data processing routines, as different software were used to process the raw data from each instrument. Because of that, a direct comparison between each image pair was performed. Since few images, from both M4 Tornado and the modular scanner, were too noisy, blurred or with a low dynamic range, they had to be first filtered to render this operation possible, otherwise the mean-squared error test would yield biased information. Furthermore, each pixel represents a conversion from the images’ respective element net-peak area to a greyscale (0–255) intensity value, requiring that few images were normalized beforehand. Finally, each image pair had its histogram corrected. This was performed by applying a histogram-matching algorithm followed by a linear contrast stretching one. Rotation corrections were dismissed, as the images already present a good agreement in this regard. The mean-squared error (MSE) and structural similarity index (SSIM) scored for each pair is given in Table 1.

SSIM algorithm measures the *quality* of an image in comparison to a reference one, working closer to the human visual system instead of the mathematically defined methods (as MSE) [25,26]. SSIM scores rate how similar two images are, 0 meaning entirely different images, and 1 meaning the two images are identical. As for MSE values, lower values mean there is theoretically less difference between the two images. Equation (1) shows how the MSE is calculated, where n is the total number of pixels, and $y_{0}$ and $y_{1}$ are the images *i*th-pixels. Detailed results can be found in the Supplementary Material Figure S1.

(1) $$MSE=\frac{1}{n}\sum_{i=1}^{n}{\left(y_{0}-y_{1}\right)}^{2}.$$

Arsenic images are considerably noisy, and a comparison between them would clearly yield unsatisfactory results for both MSE and SSIM, despite matching their histograms. The concomitant presence of Pb and As makes it hard to obtain a clear image from As-$K_{\alpha }$ line, as a significant overlapping with Pb-$L_{\alpha }$ line occurs. The obvious option is to create an image from As-$K_{\beta }$ line, which is clearly resolved but with significant less counts, due to both its low content in the sample and the inherent lower probability of K-beta emissions over K-alpha.

Most images present a low SSIM score (from 0.11 to 0.26), in particular Sn and Pb images. The structural similarity test, in fact, tends to yield a low score when comparing blurred images. For example, a same image, compared with its highly interpolated and enlarged version, will score poorly on the SSIM test [25] (Supplementary Material Figure S1l). For these images, the MSE works better as a comparison parameter.

From all elemental distribution maps obtained, the most discrepant are Sn-$K_{\alpha }$, Sb-$K_{\alpha }$, and Pb-$M_{\alpha }$. The former being close to unreadable regarding the results from both instruments. M4 Tornado’s Sn- and Sb-$K_{\alpha }$ images are entirely blurred, and the Modular Scanner Pb-$M_{\alpha }$ is completely noisy. When taking into consideration that the Modular Scanner was capable of producing an image for Sb-$K_{\alpha }$ and a better image for Pb-$L_{\alpha }$, while M4 Tornado produced better images for Sn- and Sb-$L_{\alpha }$, and Pb-$M_{\alpha }$ lines, one can clearly see where the optimal working condition of each instrument lies.

**Figure 4:**
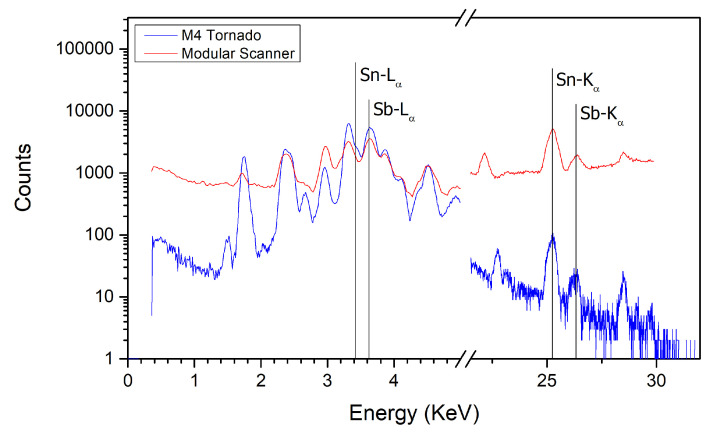
Sum spectra of the scanned regions.

The use of a polycapillary lens provided a much narrower beam, at the cost of sacrificing resolving power at higher energies, a phenomenon better described elsewhere [27]. Furthermore, Sn and Pb are expected to be present in higher quantities in the vitreous base layer, from which the signal is considerably attenuated by the superimposing pigments strata. A less intense excitation beam would highlight this factor even more, as it can be seen in Figure 4. Even though M4 Tornado sum spectrum presents narrower peaks for Sn- and Sb-$K_{\alpha }$ lines, with apparently much less background contribution at the higher energy end of the spectrum, the count-rate observed was insufficient to produce clear images.

Regarding the lower energy end of the spectra, M4 Tornado outperforms the Modular Scanner, providing a clearer image for Sb-$L_{\alpha }$ and overall better images for Sn-$L_{\alpha }$ and Pb-$M_{\alpha }$. Figure 4 shows that indeed the M4 Tornado provides a better energy resolution, signal-to-noise ratio and count-rate at the very low end of the spectrum. From mid to high energy range (≈5 to 30 KeV), the use of a collimator provided better results, with higher counts, and, therefore, better images; for example, those of Sb-$K_{\alpha }$ and Pb-$L_{\alpha }$.

For Sn and Sb, the use of polycapillary lenses can justify the loss of excitation intensity in the mid-to-high energy interval to gain on resolution, yielding more defined images at the lower energy range (L-lines). As for the remaining images, advantages provided by a smaller beam size, at the given analysis conditions, were not fully exploited. Co and Fe images, for example, have all fine details visible, for both instruments. Therefore, for larger and faster scans, where the beam size if often wider (≈1 mm), the costs trade-off between having an instrument with collimator optics rather than polycapillary lenses can be fully justified. Furthermore, collimators dismiss the need of a z-axis focusing system, as the output beam is less divergent and the irradiation spot is almost independent from the tube distance [27].

In any case, regardless of the optics chosen, the elemental distribution maps provided by the MA-XRF technique already yielded some relevant information from which it is possible to layout a multi-analytical strategy. The *azulejo* in question is clearly a majolica style tile, where coloured oxides were applied over a white lead-tin glaze. The layers disposition can first be assumed by interpreting the K- and L-lines or L- and M-lines of a same element, as the case of Sn, Sb, and Pb.

To further understand the pigments and technique used in its manufacture, complementary techniques as Raman and Monte Carlo simulations were used. The different pigments and layers are analysed individually in the following subsections.

### 3.2. White Glaze

The white, vitreous phase, that characterises the *majolica* technique, is commonly composed of Pb oxides, and Sn and Si dioxides [1]. Lead oxide is usually used to lower the siliceous matrix melting point, while tin dioxide is used as an opacifying agent. The resulting mixture (phase) can be porous and have a considerable amount of inclusions [1,5].

The elemental distribution maps, shown in Figure 3, indicate that the white glaze, present in the *azulejo* herein analysed, has lead and tin in its composition, in accordance to other white glazes observed in *azulejos* from the same period. Furthermore, with Monte Carlo simulations, it was possible to estimate this layer’s thickness, which varies from 280 to 350 μm, also compatible with previously reported tiles [1]. Raman spectra confirmed the presence of cassiterite (SnO${}_{2}$), evidenced by bands at 635 and 775 cm${}^{-1}$, a compound normally used as an opacifying agent. Simulation spectra can be found in the Supplementary Material.

### 3.3. Blue Colour

Closely observing the elemental maps in Figure 3, it is possible to notice a strong correspondence between cobalt, nickel, iron, and arsenic. The latter being an indication of the cobalt oxide origin, and iron, having a correlation that is predominant in the leftmost part and around the central S-shaped region. This quaternary combination can be directly related to *zaffre*, imported by Portugal from Germany in the beginning of the 16th century [1]. The information from the elemental maps alone gives a hint on the pigment used to obtain the blue colour, although a clear distinction between the light and dark shades was not achieved.

**Figure 5:**
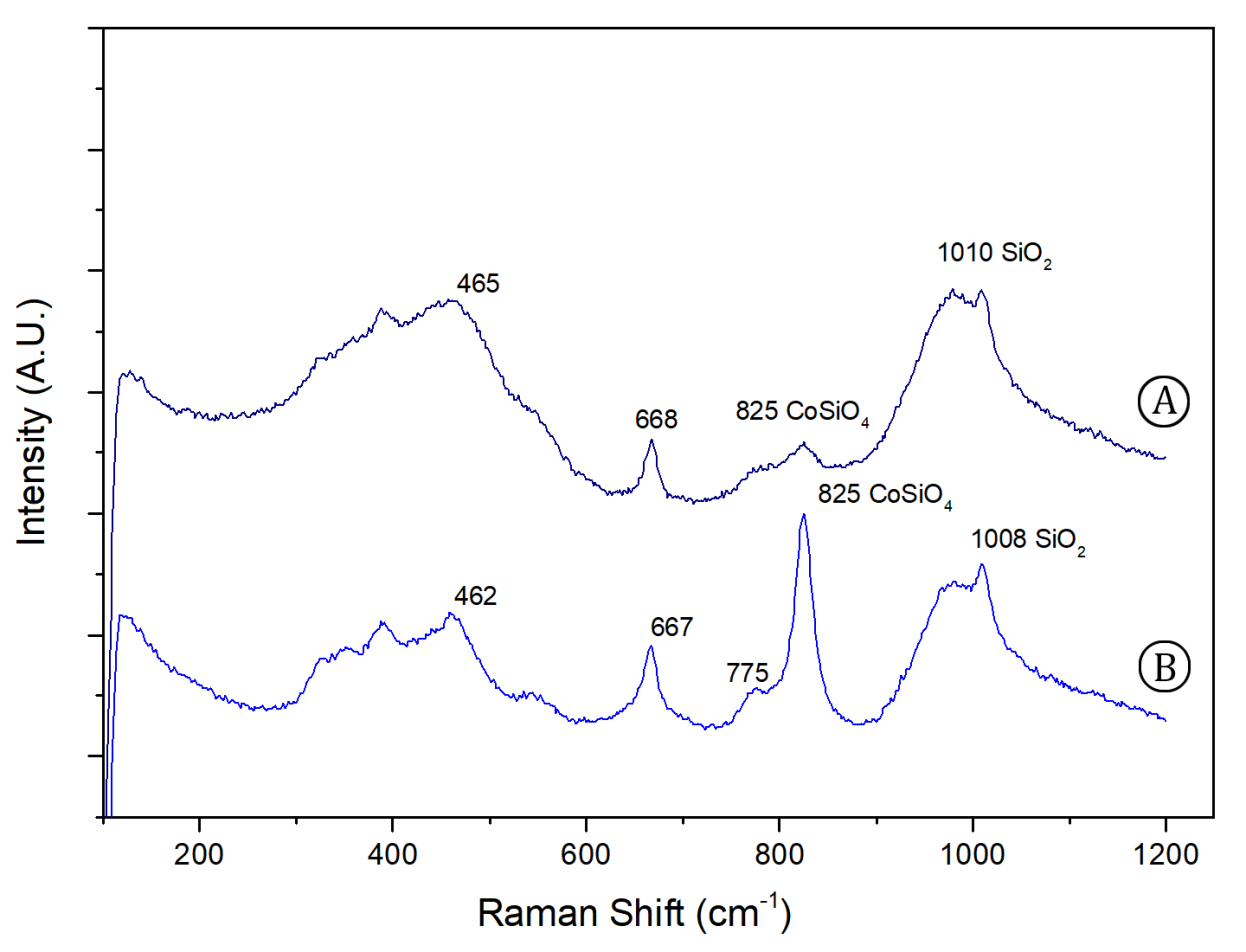
Recorded μ-Raman spectra for blue glazed regions: (**A**) vitreous blue region, (**B**) darker blue region; (**B**) $\lambda_{excitation}$ = 532 nm.

Figure 5 shows the μ-Raman spectra obtained for the blue glazed regions, which seems similar at first sight, besides representing different intensities of blue. One is a dark, intense blue, and the other, a lighter blue with glassy appearance. This difference could be explained due to the presence of a Raman band peaking at 824 cm${}^{-1}$ that allows a clear identification of a cobalt blue pigment: cobalt silicate (CoSiO${}_{4}$), olivine type, which has been previously reported by de Waal [28] and, recently, Ferreira [29].

The blue colour was traditionally obtained from cobalt oxides (CoO${}_{x}$) which dissolve in the glassy phase (SiO${}_{2}$). Co ions interact with the Si-O network and create new Si-O-Co bonds by replacing Si in the glass structure. In the 1st half of 17th century, this technique was done by dissolving cobalt ore in low quantities into the glaze, which does not necessarily lead to the precipitation of the silicate. As expected, the 824 cm${}^{-1}$ signature band is not observed for blue pigments in ceramics of this period [30]. On the other hand, in the 2nd half of the 17th century, this signal is detected, which leads to the assumption that the glazing technique was done using higher temperatures [28,31].

The difference between the blue glazed regions is probably due to the content of Co in the structure. In the vitreous blue region (Figure 5A) a lighter blue is observed, due to a lower presence of the cobalt pigment. So, there is a predominance of the glassy silicate structure, which can be detected by the Si–O stretching and bending phases (envelopes at 980 and 465 cm${}^{-1}$). In the darker blue region (Figure 5B), as expected, the 824 cm${}^{-1}$ is far more noticeable, as observed by Pereira et al. [31]. The difference in the relative amount of cobalt, for the bright and dark shades of blue, can also be observed in the elemental maps shown in Figure 3, where a more intense signal for this element is noticed in the rightmost part, together with arsenic.

Furthermore, Monte Carlo simulations of XRF spectra obtained from the blue colour, yielded a thickness of about 70 μm for both shades, light and dark. The difference relied in the chemical composition, where the dark blue presented from 2 to 4 times more iron, nickel, cobalt and arsenic. This supports the other information obtained, showing that the dark blue shade has a higher concentration of cobalt and associated elements.

### 3.4. Yellow Colour

**Figure 6:**
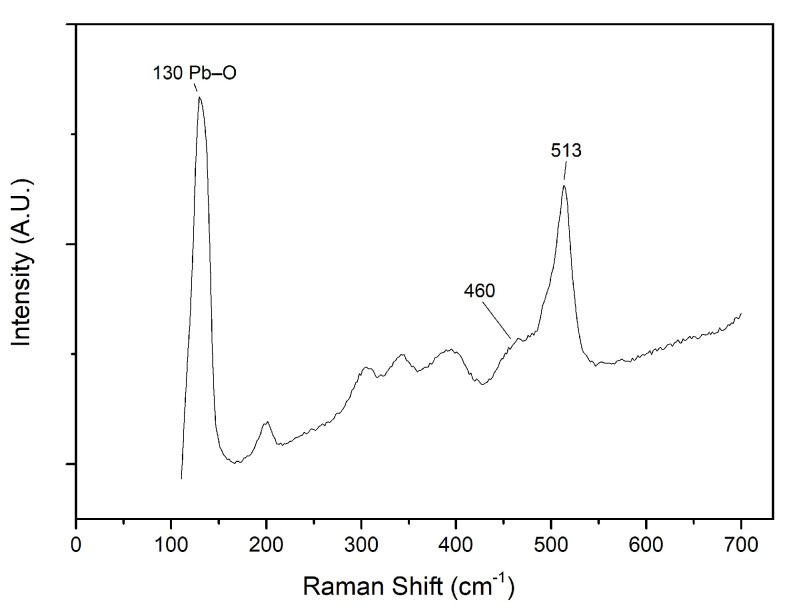
Recorded Raman spectra for a yellow glazed region ($\lambda_{excitation}$ = 532 nm).

The elemental distribution maps show the presence of Zn, Pb, and Sb in the yellow regions (Figure 3), suggesting the use of Naples Yellow ($Pb_{2}Sb_{2}O_{7}$) or a Zn-modified version of this pigment. To verify this hypothesis, the μ-Raman spectra (Figure 6) were used. The yellow-region spectra do not exactly match with the reference spectra for Naples Yellow (peaks at 136, 328, 453 and 510 cm${}^{-1}$), indicating indeed the possibility of a ternary oxide structure. Several studies [32,33,34] draw attention for these ternary oxides, as variations from the standard Naples Yellow by the addition of either Zn and/or Sn, replacing some of the Sb in the pigments structure [33] and thus creating Sn-Pb-Sb and/or Zn-Pb-Sb pyrochlores.

The very strong band at 130 cm${}^{-1}$ is reported for the Pb-O lattice vibration at low wavenumbers (120–139 cm${}^{-1}$), and should not be used as a good discrimination band for ternary oxides. Furthermore, it varies significantly even for unmodified Naples Yellow and different firing temperatures for the pigment [34]. It is worth noting that for Zn modified Naples Yellow, this band usually appears at 145 cm${}^{-1}$, while for the Sn modified, it shows a doublet at 125 and 142 cm${}^{-1}$ [33].

In unmodified Naples Yellow there is a strong band at 513 cm${}^{-1}$ for the $SbO_{6}$ octahedra symmetric elongation. This band is much more useful for discrimination and characterization of ternary oxides, since it collapses and shows another band at ca. 450 cm${}^{-1}$ for modified pigments [1]. Even though the collected spectrum shows a band at ca. 460 cm${}^{-1}$, it is worth noting that this band can present variations with the type of ternary oxide and composition. Nonetheless, is can still be used as a good indicator of the presence of ternary oxides.

Furthermore, accordingly to Sandalinas et al. [34], for Sn-Pb-Sb pyrochlores there should be a band at ca. 640 cm${}^{-1}$, which could not be detect in the obtained spectra for the yellow region. This further reinforces the possible presence of a Zn-modified Naples Yellow.

Finally, Monte Carlo simulations revealed the presence of a multi-layered structure in the yellow region, demonstrating that the yellow pigment was painted on top of the white glaze, according to the *majolica* technique, and with a thickness of about 50 μm. The composition of the yellow strum, in the simulations, revealed a high content of lead and antimony, as expected and demonstrated by both the Raman spectra and elemental distribution maps. Zinc, on the other hand, was found in low percentages within the simulations, but present nonetheless.

### 3.5. Orange Colour

When observing Pb-$L_{\alpha }$ and Fe elemental distribution maps (Figure 3), one can notice an absence of correlation between these two elements in what concerns the orange region (resembling an S-shape). However, in an opposite way, Zn and Sb present a correlation with the orange region and the Pb-$M_{\alpha }$ image, analogously to the yellow region. From the Pb images, it is possible to assume that lead oxides are also contained within the orange matrix. This indicates an intentional addition of iron oxides (possibly rust [35]) to the ternary yellow pigment in order to obtain the observed orange hue.

**Figure 7:**
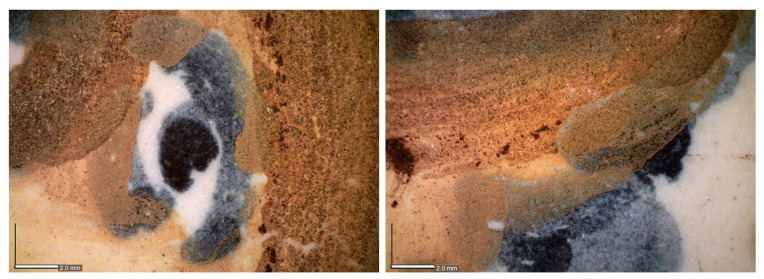
Micrographs from the yellow, blue and orange colours. Scale bar is 2 mm wide.

Analysing the orange colour under a microscope, the pigment reveals itself more similar to the yellow one indeed, but rougher, with the presence of dark spots, and without the characteristic shiny and transparent layer present on top (Figure 7). This last stratum (*coperta*) is a very thin layer of powdered glass sprinkled on top of ceramics in order to conceive a shiny and smooth aspect to the final product [35].

**Figure 8:**
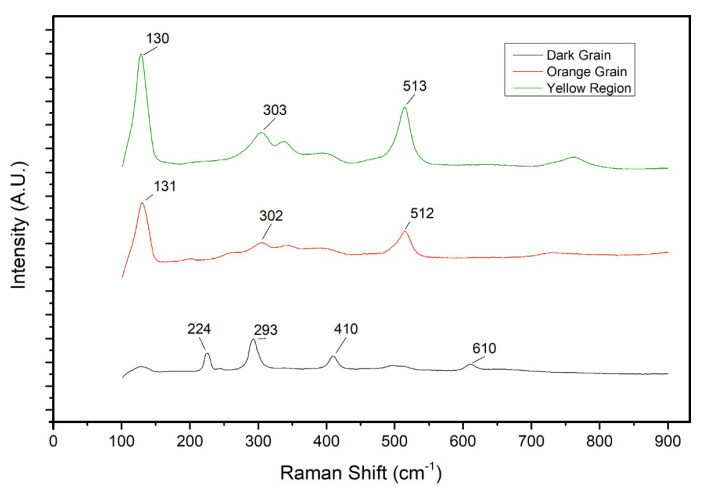
Comparison of recorded Raman spectra obtained from the orange region: orange grain, yellow matrix, and dark grain. The $\lambda_{excitation}$ for the dark grain spectrum was of 785 nm, while for the remaining spectra, 532 nm.

To further understand the pigments’ nature, μ-Raman spectra (Figure 8) were analysed. They appear to be very akin to the spectra obtained from the yellow region, showing similar bands and peaks (at ca. 131, 302 and 512 cm${}^{-1}$) and indicating the use of a mixture obtained from the modified Naples Yellow and other components, as previously seen in the yellow colour.

The recorded spectrum for a dark grain (Figure 7) observed within the orange colour composition (Figure 8) shows very visible bands at ca. 293, 410 and 610 cm${}^{-1}$, which matches with α-hematite (Fe${}_{2}$O${}_{3}$) reference spectrum [30]. This can be an indicative that the modified Naples Yellow was indeed mixed with hematite to obtain the desired orange colour. This hypothesis can be partially supported by the strong presence of Fe in the XRF spectra for the orange region and previous studies on orange colours used on *azulejos* from the same period [1,4].

Monte Carlo simulations did not yield satisfactory results for this particular pigment, as the stratigraphy appears to be relatively over-complex and there was not much conclusive information (possible layering structure and layers composition) to use as an initial guess to feed the model.

## 4. Conclusions

The *in-house* developed modular MA-XRF scanner proposed had its performance compared to a commercial instrument. The results obtained from both instruments were compared and discussed. A clear difference in resolution, in the lower energy end of the spectra, probably thanks to the more expensive, sub-mm X-ray optics and X-ray detector, used in the commercial system could be observed. However, for general purposes, larger and faster scans, the use of cost-effective optics could be justified.

Moreover, not only did the proposed instrumentation performance proved satisfactory but also it demonstrated to be a good alternative to expensive and bulky commercial instrumentation. The modular scanner can be faced as a fully portable unit or a mobile instrument, performing similarly to fixed instruments under similar analysis conditions.

Elemental distribution maps obtained from both scanners were combined with two complementary analytical techniques, to thoroughly investigate a 17th century Portuguese *azulejo*. The overall stratigraphy of the sample, as well as the identification of most colouring agents was possible with MA-XRF data alone. Raman analysis was fundamental in understanding the differences in the shades of blue found in the sample as well as confirming the yellow pigment hypothesis. Monte Carlo simulations proved to be a powerful tool, determining the stratigraphy, composition and thickness of almost all pictorial layers.

In summary, the pigments identified in the *azulejo* were: (a) cobalt blue (obtained from *Zaffre*) in varying concentrations to obtain different shades of blue, (b) Zn-modified Naples Yellow, and, (c) a combination of hematite and Zn-modified Naples Yellow, to obtain the orange colour. The white glaze was found to be a silica (SiO${}_{2}$) matrix, modified with the use of lead oxide (flux) and tin dioxide (opacifier).

## Figures and Tables

**Table 1:** Image scores between the Modular Scanner and M4 Tornado images.

	$K_{\alpha }$		$K_{\beta }$		$L_{\alpha }$		$M_{\alpha }$	
**Element**	**MSE**	**SSIM**	**MSE**	**SSIM**	**MSE**	**SSIM**	**MSE**	**SSIM**
Fe	539.30	0.71	-	-	-	-	-	-
Co	556.82	0.68	-	-	-	-	-	-
Ni	678.35	0.44	-	-	-	-	-	-
Zn	781.04	0.40	-	-	-	-	-	-
As	-	-	1116.47	0.27	-	-	-	-
Sn	1673.41	0.11	-	-	1957.48	0.11	-	-
Sb	2194.59	0.21	-	-	1167.72	0.50	-	-
Pb	-	-	-	-	146.66	0.14	2883.70	0.12

## Data Availability

The data presented in this study are available upon request to the authors.

## Supplementary Materials

The following are available online at https://www.mdpi.com/1424-8220/21/5/1913/s1, Figure S1: Detailed SSIM and MSE comparisons, Figure S2: Full sum spectrum of scanned region, Figures S3–S6: Monte Carlo simulated and experimental spectra comparisons, Table S1: Monte Carlo layers final composition, Video S1: MA-XRF scanning 3D render.
