# Supplementary material for: Modular MA-XRF Scanner Development in the Multi-Analytical Characterisation of a 17th Century Azulejo from Portugal
Source: Sensors (Basel). 2021 Mar 9;21(5):1913. doi: 10.3390/s21051913 (PMC7967215; doi:10.3390/s21051913)
Supplement: Supplementary file 1 [file sensors-21-01913-s001.zip › Supplementary Material 1.pdf]

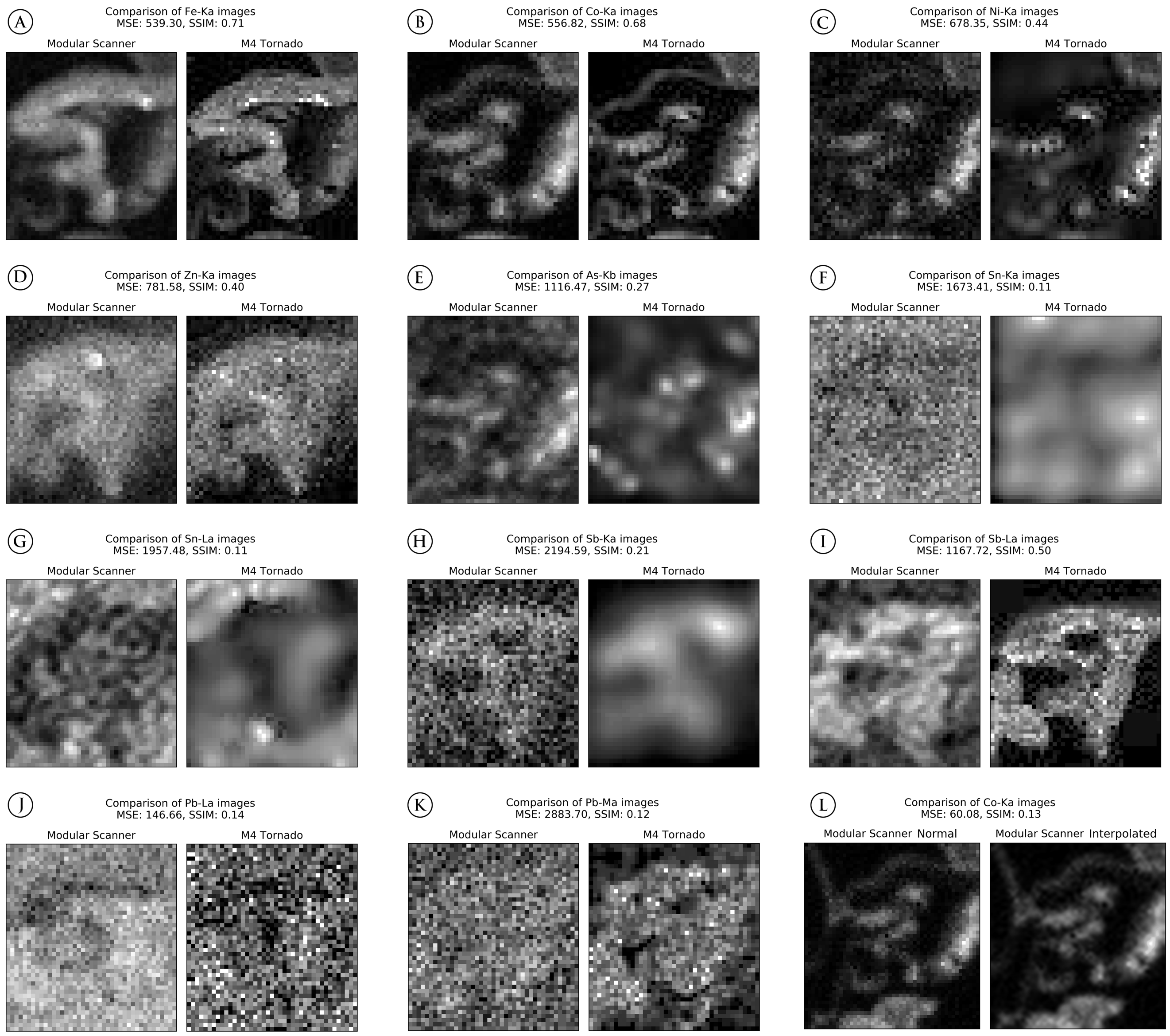

Figure 1: Comparison scores between the Modular Scanner and M4 Tornado images (A-K), and comparison between a same image, interpolated from 50 x 53 px^2. to 1024 x 1085 px^s with nearest neighbour (normal) and bicubic (interpolated) algorithms (L).

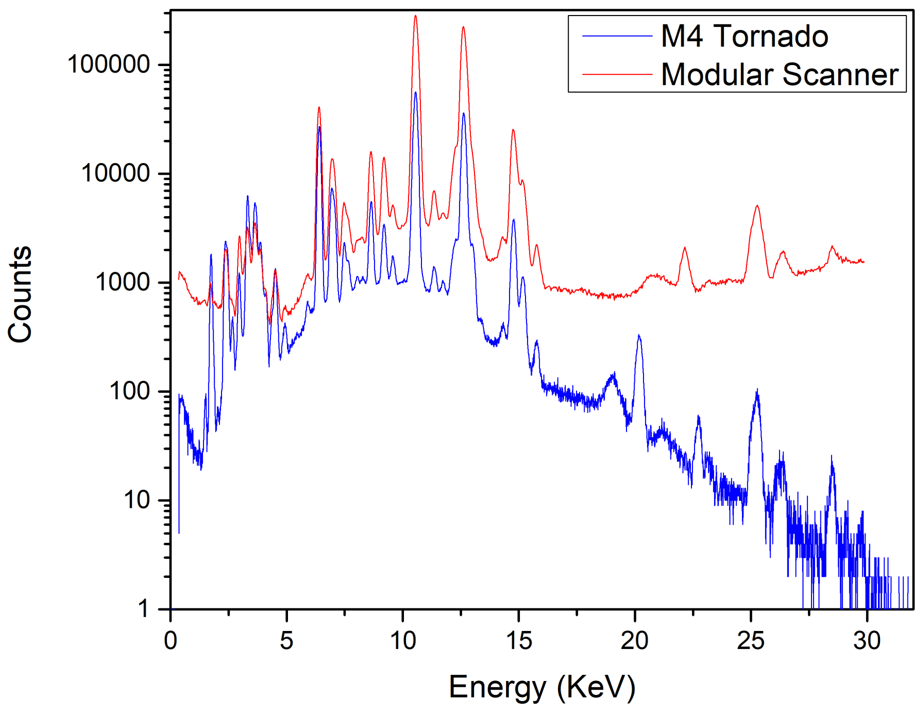

Figure 2: Sum spectra of the scanned regions.

Spectrum	Maximum scattering order	Multiplicity of events (Order 1, 2, ..., n)	Layers Count
Ceramic + White	3	200, 200, 200	2
Dark Blue	3	200, 200, 200	3
Light Blue	3	200, 200, 200	3
Yellow	3	200, 200, 200	3

Table 1: Photoelectric order parameters and layer numbers.

Spectrum	Layers thickness (from bulk to surface)		
	Layer 1	Layer 2	Layer 3
Ceramic + White	0.89 cm	280 um	-
Dark Blue	0.86 cm	280 um	70 um
Light Blue	0.86 cm	280 um	70 um
Yellow	0.87 cm	280 um	50 um

Table 2: Layers thicknesses.

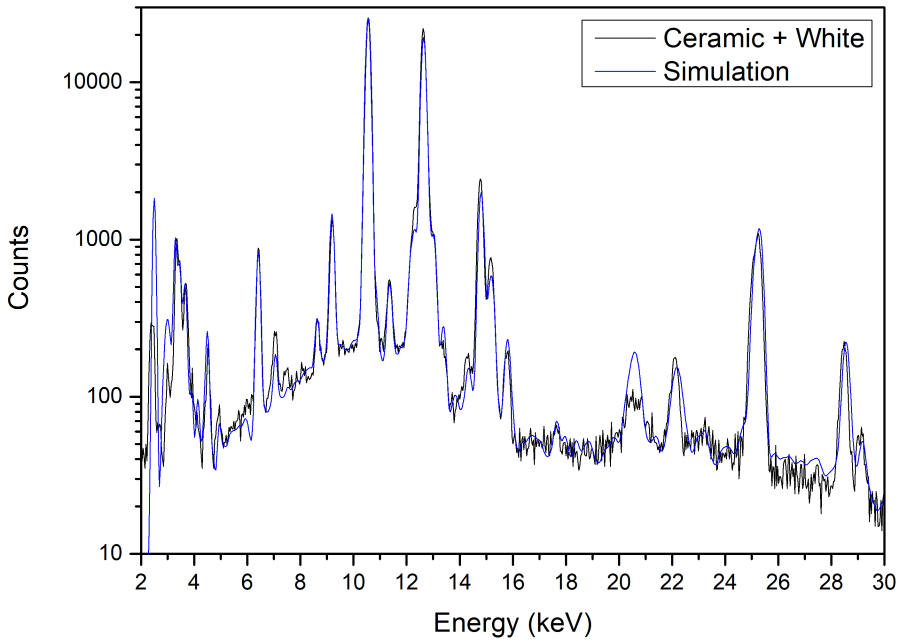

Figure 3: Monte Carlo simulation spectrum and experimental spectrum for the ceramic plus white glaze region.

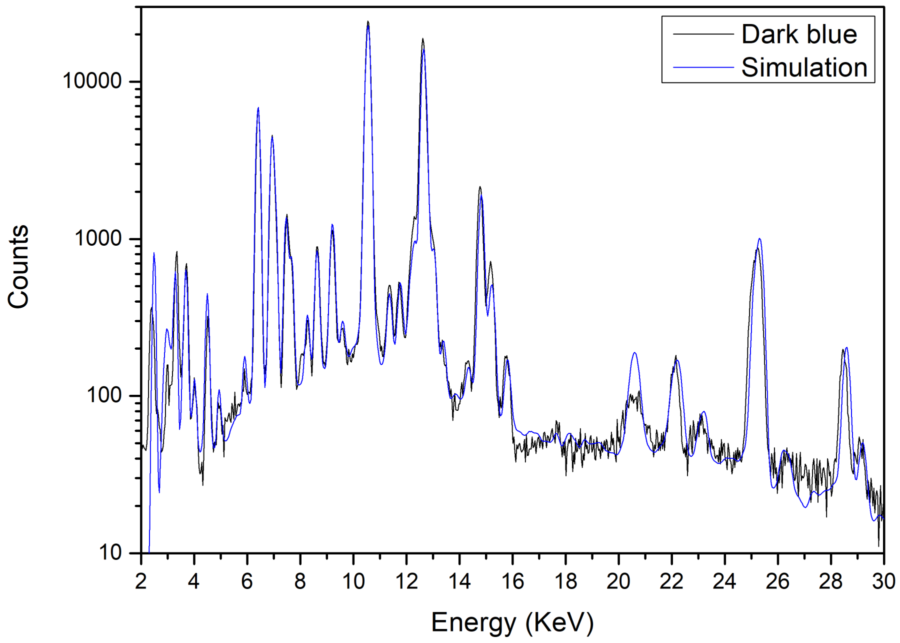

Figure 4: Monte Carlo simulation spectrum and experimental spectrum for the dark blue colour shade.

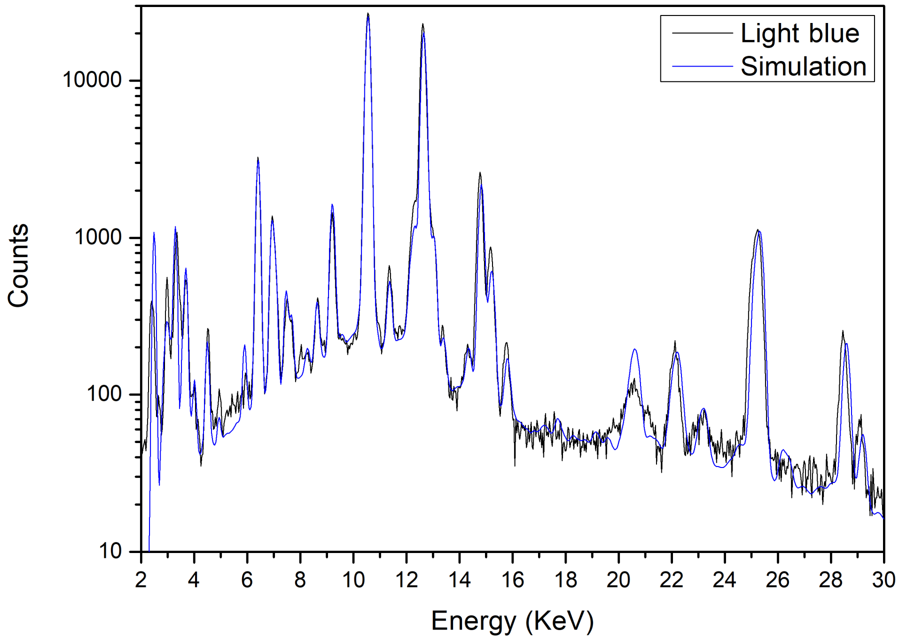

Figure 5: Monte Carlo simulation spectrum and experimental spectrum for the light blue colour shade.

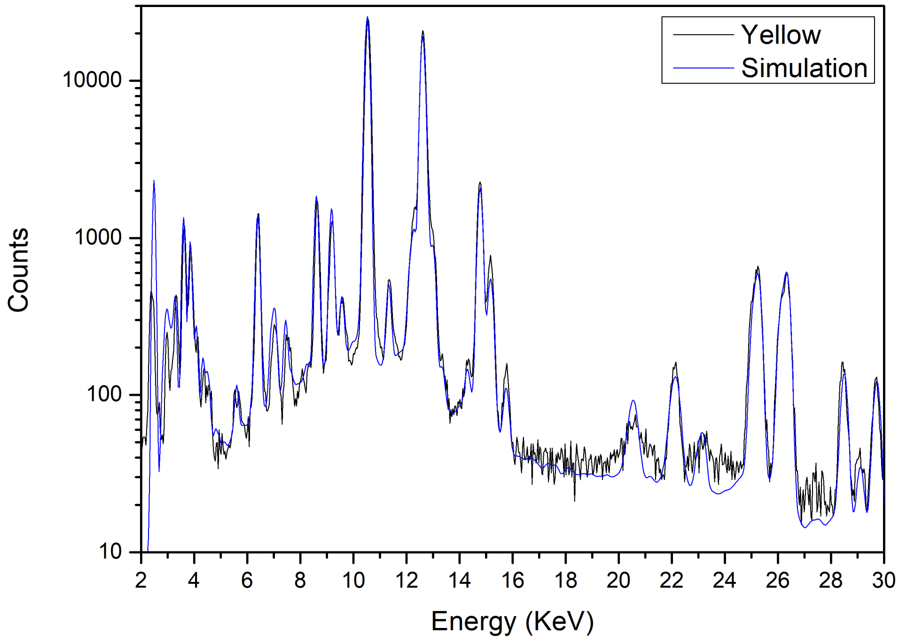

Figure 6: Monte Carlo simulation spectrum and experimental spectrum for the yellow colour.
